# Changes in cortical network connectivity with long-term brain-machine interface exposure after chronic amputation

**DOI:** 10.1038/s41467-017-01909-2

**Published:** 2017-11-27

**Authors:** Karthikeyan Balasubramanian, Mukta Vaidya, Joshua Southerland, Islam Badreldin, Ahmed Eleryan, Kazutaka Takahashi, Kai Qian, Marc W. Slutzky, Andrew H. Fagg, Karim Oweiss, Nicholas G. Hatsopoulos

**Affiliations:** 1 Department of Organismal Biology and Anatomy, University of Chicago, Chicago, IL 60637 USA; 2 Committee on Computational Neuroscience, University of Chicago, Chicago, IL 60637 USA; 3 School of Computer Science, University of Oklahoma, Norman, OK 73019 USA; 4 Department of Electrical & Computer Engineering, University of Florida, Gainesville, FL 32611 USA; 5 Department of Electrical & Computer Engineering, Michigan State University, East Lansing, MI 48824 USA; 6 Department of Biomedical Engineering, Illinois Institute of Technology, Chicago, IL 60616 USA; 7 Departments of Neurology, Physiology, and Physical Medicine and Rehabilitation, Northwestern University Feinberg School of Medicine, Chicago, IL 60611 USA; 8 Present Address: Department of Neurology, Northwestern University, Chicago, 60611 IL USA

## Abstract

Studies on neural plasticity associated with brain–machine interface (BMI) exposure have primarily documented changes in single neuron activity, and largely in intact subjects. Here, we demonstrate significant changes in ensemble-level functional connectivity among primary motor cortical (MI) neurons of chronically amputated monkeys exposed to control a multiple-degree-of-freedom robot arm. A multi-electrode array was implanted in M1 contralateral or ipsilateral to the amputation in three animals. Two clusters of stably recorded neurons were arbitrarily assigned to control reach and grasp movements, respectively. With exposure, network density increased in a nearly monotonic fashion in the contralateral monkeys, whereas the ipsilateral monkey pruned the existing network before re-forming a denser connectivity. Excitatory connections among neurons within a cluster were denser, whereas inhibitory connections were denser among neurons across the two clusters. These results indicate that cortical network connectivity can be modified with BMI learning, even among neurons that have been chronically de-efferented and de-afferented due to amputation.

## Abstract (web summary)

Previous studies have shown short-term plasticity in single neurons or local field potentials during brain-machine interface (BMI) training. Here the authors report long-term changes in functional connectivity of motor cortex neuronal ensemble activity as chronically amputated monkeys learn to operate a BMI.

## Introduction

Exposure to a brain–machine interface (BMI) provides a useful paradigm for examining the relationship between neural plasticity and motor skill acquisition because a BMI creates a causal relationship between a subset of recorded neurons and the behavioral output of the device being controlled. Therefore, any behavioral learning that takes place in a BMI paradigm must be due to changes in neural activity among the recorded neurons, although plasticity in neural activity may be due to synaptic plasticity occurring outside the recorded area. A number of BMI studies have shown short-term (within a daily session) and long-term (across days) learning^1–3^. Associated changes in modulation and tuning curves of single cells and local field potential oscillations have also been observed as BMI learning takes place^1, 3–10^. However, much less, if any, work has examined changes in network connectivity associated with BMI experience.

Most BMI studies that have examined neural plasticity and learning have faced two limitations. First, typical BMI experiments recalibrate the decoders from day to day because neurons are lost or gained due to recording instabilities. Therefore, both the decoder and the recorded neural population vary over time. To isolate the effects of neural plasticity on BMI learning, it is important to keep the decoding parameters fixed and to record from the same population of neurons over long-term exposure. Second, most BMI studies involving non-human primates have relied on healthy, intact animals or animals that have undergone acute nerve blocks^11^ (however, see ref.^12^ for a brain–spine interface to by-pass spinal lesions). Therefore, it is unclear whether observed neural plasticity can generalize to patients with severe and often chronic motor disabilities such as spinal cord injury or amputation who are the ultimate recipients of BMI systems.

To address these limitations, we used fixed decoders with a subset of neurons that were stably recorded over a one month BMI learning experiment. Moreover, we used unilaterally amputated monkeys that had undergone therapeutic amputation several years (two of them with 9 to 10 years and a third monkey over 4 years) before they arrived in our lab. The use of chronically amputated monkeys is arguably the most clinically-relevant model of human amputation and an important model of chronic de-afferentation and de-efferentation where reorganization of motor and somatosensory maps in cortex has taken place^13–16^.

We implanted multi-electrode arrays in the upper limb area of primary motor cortex (MI) either contralateral (monkeys Z and N) or ipsilateral (monkey K) to the amputation. We then arbitrarily assigned two clusters of recorded neurons to control either the reaching or grasping velocities of a multiple degree-of-freedom robot and trained the monkeys to volitionally modulate these clusters of neurons to perform a sequential movement consisting of reaching toward a target object, grasping it, pulling it back, and finally releasing it. Applying a statistical causality analysis to the network of neurons used to control the robot, we determined whether network connectivity in the “contralateral” monkeys would emerge among neurons that had not controlled an intact limb for several years and compared the evolution in network connectivity with that observed in the “ipsilateral” monkey whose motor cortex continued to control an intact limb.

## Results

### BMI learning

**Fig. 1:**
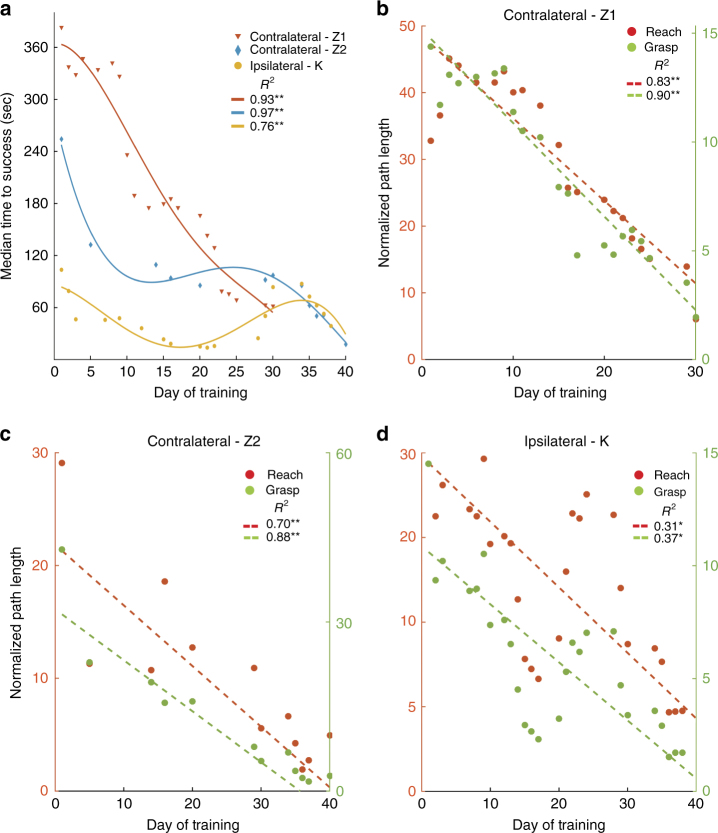
Behavioral performance of the subjects. **a** shows the median time to success over BMI training. Time to success refers to the time taken to perform a successful trial of the reach-grasp-pull-release task. The discrete points in the plot correspond the actual data, and the solid lines show a fourth order polynomial fit to the data. **b**–**d** shows the improvement in mean normalized path lengths of reach and grasp movements. Normalized path length refers to the total path traveled by the robotic arm to complete a successful trial divided by the shortest path possible. The dotted lines represent least-square fit for the data (shown as discrete points). **p < 0.001 and *p < 0.01

The subjects learned to control the reach and grasp velocities of the robot through volitional modulation of spiking activity to perform the sequential reach-to-grasp task. The task was self-paced with no preset time limit to successfully perform a trial. The median time to successfully perform a trial decreased with BMI exposure from ~370 to 60 s and ~250 to 20 s for the two experiments with the contralateral monkey Z, from ~30 to 21 s for the contralateral monkey N and from ~110 to 40 s for the one experiment with the ipsilateral monkey K (for monkeys Z and K see Fig. 1a; for monkey N see Supplementary Fig. 1b (left panel); solid lines show a fourth order polynomial fit to the data points; for monkey N, the fit was a third order polynomial fit). We also assessed the efficiency in performing the task by measuring the normalized path length defined as the total path length traversed by the hand (reach) or aperture (grasp) divided by the shortest path possible without path reversals. The mean normalized path lengths of the reach and grasp control dimensions decreased for the two experiments with the contralateral monkey Z (Fig. 1b–c; dotted lines show least-square fit for the data points; contralateral-Z1 *R* ^2^ = 0.83 (reach, *p* < 0.001; t-statistic with *n *= 21) and 0.90 (grasp, *p* < 0.001; t-statistic with *n* = 21); contralateral-Z2 *R* ^2^ = 0.70 (reach, *p <* 0.001; t-statistic with *n* = 12) and 0.88 (grasp, *p* < 0.001; t-statistic with *n* = 12)) and for the single experiment with the contralateral monkey N (Supplementary Fig. 1b (right panel); *R* ^2^ = 0.67 (reach, *p* < 0.01; t-statistic with *n *= 5) and 0.07 (grasp, *p* ~ 0.1; t-statistic with *n* = 5)). Likewise, for the ipsilateral monkey K, the normalized path lengths decreased with BMI exposure (Fig. 1d; Ipsilateral-K *R* ^2^ = 0.31 (reach, *p* < 0.01; t-statistic with *n* = 20) and 0.37 (grasp, *p* < 0.01; t-statistic with *n *= 20)). Although the three animals (Z, N and K) exhibited learning effects, their movement strategies were different such that monkey Z tended to perform reaching and grasping in a sequential manner (see Supplementary Movie 1 for qualitative demonstration and Supplementary Movie 2 for improvements with learning), whereas monkey K and N exhibited more simultaneous coordination of the reaching and grasping movements.

### Emergence of effective connectivity

**Fig. 2:**
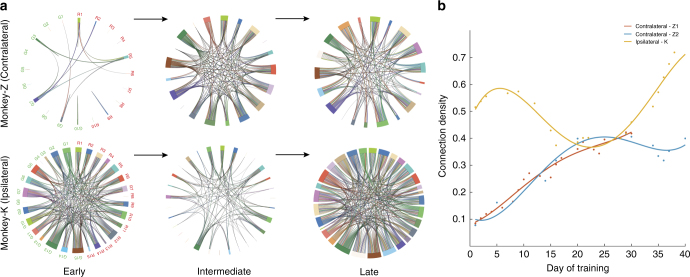
Network density in the neuronal populations. (**a**, Top) Three instances along the days of training shows a monotonic increase in network connectivity in the contralateral monkey Z (contralateral-Z2). Each chord represents a connection between two neurons. (**a**, Bottom) shows the connectivity in the ipsilateral monkey K. There was an initial decrease in the network density followed by an increasing phase. Different colored chords represent individual neurons. The labels R_n_ (red) and G_n_ (green) correspond to the reach and grasp neurons, respectively. **b** The connection density along the entire course of training for two experiments with monkey Z and one experiment with monkey K

We developed a statistical model to predict the current spiking activity of each neuron based on the spike history of all neurons in the reach and grasp clusters (see Methods section). Using this model, we then determined statistically significant directed connections among all neuron as BMI learning took place. The temporal evolution of connection density (i.e., number of connections divided by the total number of possible connections) with BMI learning was quite distinct between the contralateral and ipsilateral monkeys (Fig. 2a; Supplementary Fig. 2). Both contralateral monkeys began with a sparse network early in BMI exposure whose connection density increased in a nearly monotonic fashion with BMI learning (Fig. 2b, red and blue lines for contralateral-Z1 (with Decoder A) and contralateral-Z2 (with Decoder B), respectively; Supplementary Fig. 3a for monkey N with Decoder B). In contrast, the ipsilateral monkey began with a dense network early in BMI learning, which was then pruned in the middle of BMI learning, and then increased in connection density at the end of BMI learning beyond the initial density (Fig. 2b, yellow line; ipsilateral-K with Decoder B).

To more closely examine the dynamics of network connectivity, we parsed the entire network into four subnetworks comprising (1) directed connections between the neurons within the reach and (2) grasp clusters, (3) directed connections from the grasp cluster and terminating in the reach cluster and (4) directed connections from the reach cluster and terminating in the grasp cluster (Supplementary Figs. 4–6). Across all four subnetworks, we observed a similar trend in connection density as we had with the entire network where connection density increased nearly monotonically for the contralateral monkey Z, and connections were pruned followed by an increase in connectivity in the ipsilateral monkey K.

### Connection polarity

**Fig. 3:**
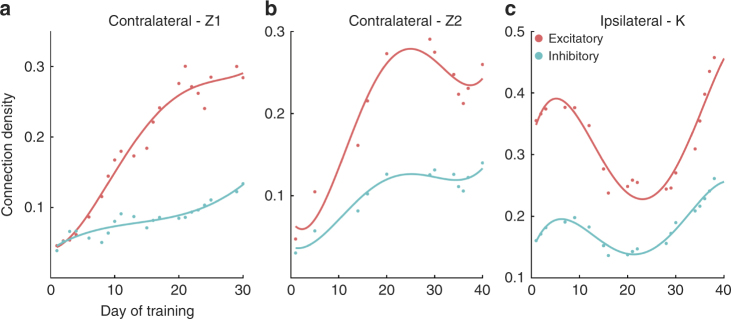
Network connection density among excitatory and inhibitory connections. **a**, **b** correspond to the two experiments with the contralateral monkey Z and **c** shows the network density in the ipsilateral monkey K. (solid lines are fourth order polynomial fits to the data points)

By examining the sign of the average value of spike history coefficients among all significant connections (see Table 1), we could compare the temporal evolution of excitatory and inhibitory connections during BMI learning. For the contralateral monkeys, both excitatory and inhibitory connection density increased although the excitatory connection density was consistently larger and increased faster reaching a level that was over 100% higher than the inhibitory connection density at the end of BMI learning (Fig. 3a, b; Supplementary Fig. 3b; see also Supplementary Fig. 7 for a third experiment with monkey Z using decoder A and a position controller where the decoded velocity was temporally integrated to generate position commands to the robot). For the ipsilateral monkey, both excitatory and inhibitory connection density exhibited pruning, and, as with the contralateral monkeys, excitatory connection density was consistently larger than inhibitory connection density (Fig. 3c).

**Table 1:** Granger causality analysis to estimate connectivity

Steps	Formulation
1. Modeling the CIF using GLM framework	$\log \lambda _i\left( {t{\mathrm{|}}\theta _i,H\left( t \right)} \right) = \theta _{i,0} + \mathop {\sum }\nolimits_{n = 1}^N \mathop {\sum }\nolimits_{m = 1}^{M_i} \theta _{i,n,m}R_{n,m}\left( t \right),$where, $\theta _{i,0}$ represents the baseline activity, and $\theta _{i,n,m}$ represents the effect of ensemble spiking history $R_{n,m}\left( t \right)$ of neuron *n* on the firing probability of neuron *i* at time t for *n* = 1, …, *N* neurons. Here $R_{n,m}\left( t \right)$ is modeled as the spiking history using a binwidth W (set as 3 ms) spanning the interval [*t – mW, t-(m-*1*)W*]
2. Selecting model order	Akaike’s Information Criterion
3. Likelihood of causality	${\mathrm{\Gamma }}_{ij} = \log \frac{{L_i(\theta _i)}}{{L_i(\theta _i^j)}}$
	$$= \log \frac{{\Pr (t + 1\,of\,i|{\rm past}\,{\rm of}\,{\rm everyone}\,{\rm upto}\,t)}}{{\Pr (t + 1\,{\rm of}\,i|{\rm past}\,{\rm of}\,{\rm everyone}\,{\rm except}\,j\,{\rm upto}\,t)}}$$
4. Connection polarity	${\rm{sgn}}\left| {\mathop {\sum }\nolimits_{m = 1}^{M_i} \theta _{i.j.m}} \right|$

**Fig. 4:**
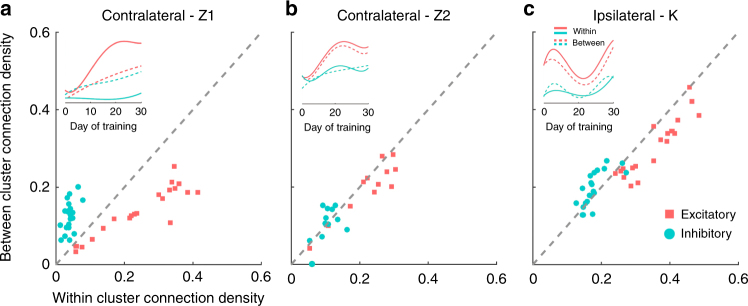
Excitatory and inhibitory connection density within and between the reach and grasp clusters. **a**–**c** The connection density within vs. across clusters for excitatory (red points) and inhibitory (blue points) connections. Each point corresponds to a given training day. Gray dashes correspond to the identity line. Inset shows the temporal dynamics of the connections resolved by clusters. Solid lines represent within-cluster connection density and dashed lines represent between-cluster connection density

We then compared excitatory and inhibitory connection density within vs. across the reach and grasp clusters across all training days. To do this, we measured the density of directed connections projecting out from a given neuron (i.e., the out-degree density) to other neurons within or across clusters. For both monkeys Z and K, excitatory connection density within clusters was significantly higher as compared to across clusters (Fig. 4a–c; see red squares, and red lines in the inset, *p* < 0.01, paired *t*-test; see Supplementary Fig. 7b for Contralateral-Z3 experiments). In contrast, inhibitory connection density was significantly higher across clusters as compared to within clusters (Fig. 4a–c; see blue circles, and blue lines in the inset, *p* < 0.05, paired *t*-test). Similar trends were observed in the reach and grasp clusters of the contralateral monkey N (Supplementary Fig. 3c), but with the limited period of BMI exposure, the differences were not statistically significant for this monkey.

### Relationship between connectivity and firing rate

**Fig. 5:**
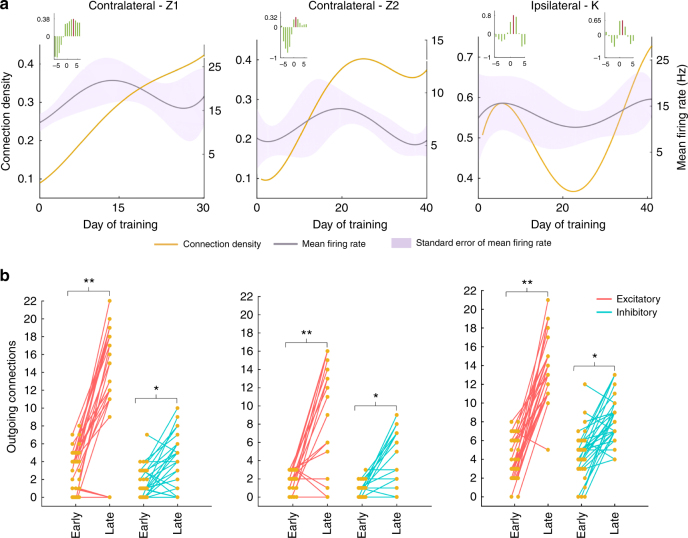
Relationship between connectivity and firing rate. **a** shows the mean firing rate (gray line; the standard error is shown as shaded region) along with the overall connectivity (yellow line). Inset shows the cross-correlation between mean firing rate and the normalized connectivity. The peak correlation is marked in red line. **b** Connection density projecting out of each neuron (i.e., out-degree density) during an early pooled data set and a late learning day is shown. The early pooled and late data sets had comparable spike counts. Each point corresponds to the number of out-degree connections of a single neuron. Red and blue lines connect excitatory and inhibitory out-degree densities, respectively. (Paired t-test, **p < 0.01, *p < 0.05)

As connectivity evolved with BMI learning, we also observed concomitant changes in firing rate among the neurons in the two clusters that mirrored the changes in connection density to a certain extent (Fig. 5a). This could be potentially problematic as our statistical model will more likely find a significant connection between neurons with high spike counts, if a functional connection exists. To discount this possibility, we first cross-correlated the dynamics in connection density with the changes in mean firing rate over BMI learning sessions. For the contralateral monkey Z, we found that the peak correlation occurred at a time lag of at least one learning session indicating that the mean firing rate modulation led the changes in connection density and suggesting that the connection density was not simply an artifact of overall firing rate (Fig. 5a (inset)). Likewise, for the ipsilateral monkey K, we found that the firing rate changes led the changes in connectivity by one training session during the initial pruning as well as during later growth in connectivity. Second, we compared connectivity in a late learning data set with the connectivity in a data set that pooled three early learning sessions so that the total spike counts in both data sets were comparable. For both monkeys, the connection density was significantly higher in the late learning data set even controlling for total spike count (Fig. 5b; paired *t*-test, *p* < 0.01 for the excitatory connections and *p* < 0.05 for the inhibitory connections).

To extend the analysis further, we classified the neurons based on the correlation between the individual firing rates and the network density. Neurons with firing rates significantly correlated to network density were formed into one group and the remaining neurons formed the second group (see Supplementary Fig. 8). For the Contralateral-Z2 neurons, both groups showed nearly monotonic increases in their connection density irrespective of the differing profiles in their firing rates. Similarly, for the Ipsilateral-K neurons, the connection dynamics remained comparable for the two groups of neurons, despite their firing rate differences.

## Discussion

We have successfully demonstrated that learning to use a cortically-controlled BMI to perform a complex, sequential task is possible in chronically amputated animals. This is significant as it is known that the motor cortex undergoes reorganization following amputation and thus has clinical implications for human amputees^17–26^. We have also demonstrated here the possibility of using ipsilateral cortex of a chronic amputee to successfully perform a BMI task and compared the dynamics of its plastic changes with contralateral networks. This has implications on developing BMIs for lateralized cortical deficit, where neural plasticity is required to accommodate movement control of both BMI and intact arm within a single hemisphere.

While the contralateral monkey Z exhibited a more dramatic decrease in median time to success, both monkeys (Z and K) managed to ultimately perform a successful sequential movement in <30 s within 12–21 training sessions. Likewise, both monkeys became more efficient in controlling the reach and grasp components of the task by reducing their normalized path length by up to a factor of 30. There was no clear indication that learning had reached asymptotic performance, so performance would have likely improved even further with extended BMI exposure.

Most studies on BMI exposure^3, 25, 26^ have used the same group of neurons to decode multiple degrees of freedom. In contrast, we used different clusters of neurons assigned to control the reach and grasp dimensions separately, and observed plastic changes occurring within and across the clusters. This approach of clustering the neurons provided a means to assess network changes occurring within and across distinct functional groups.

Neural plasticity can be a more reliable manifestation of BMI learning^27, 28^ compared to extrinsic performance metrics. First, the behavioral metrics showed fluctuations during learning (see contralateral data in Fig. 1a), while the connection density showed a near monotonic increase with exposure (Fig. 2b). By using fixed decoders with stably recorded neurons, we had the opportunity to isolate the effects of learning on neural plasticity in the same set of neurons over a 40-day period. Second, although different decoders resulted in somewhat different performance results (Fig. 1a, red vs blue), the overall dynamics of network density were very similar (see Fig. 2b, red vs blue).

Neural adaptation with BMI exposure has shown single cell plasticity including changes in the tuning properties of individual neurons^7, 8, 29^. Synchrony in a specific bandwidth of neural oscillations has also been shown to increase with BMI exposure^9^. Likewise, with operant conditioning of a neuroprosthetic device, the coherence between neural activity in M1 and dorsal striatum has been shown to increase^30^. It was previously argued that plasticity at the subcortical levels is necessary to learn neuroprosthetic skills^31^. However, further studies would be needed to establish whether subcortical plasticity is critical also for chronic amputees acquiring neuroprosthetic skills and whether such plasticity have any causal influence on the cortical plasticity.

Unlike most other studies that have examined single cell properties, we characterized the dynamics in functional connectivity in a network of M1 neurons and observed a striking difference in network plasticity between the contralateral and ipsilateral monkeys. As might be expected, the contralateral monkeys began with a sparsely connected network early in BMI learning with a normalized connection density of ~0.05 (i.e., 5%). The contralateral motor cortex had not controlled an intact limb for several years, and thus had been severely de-efferented and de-afferented for an extended time. With BMI learning, the contralateral networks steadily increased their connection density attaining an excitatory connection density of 30–40% and an inhibitory connection density of 10–20%.

The ipsilateral monkey, in contrast, began with a large connection density of nearly 40 and 20% among excitatory and inhibitory connections, respectively. Although the ipsilateral motor cortex may have experienced disrupted activity from the motor cortex on the contralateral side, it had continued to control an intact limb and had received normal afferent feedback from the intact limb which may explain why the connection density was higher than in the contralateral monkey. A number of studies have shown motor representations in motor cortex associated with ipsilateral limb movements as well as contralateral limb movements^32, 33^. Moreover, studies have used imagined or overt ipsilateral limb movements to control a BMI^34–36^. In our study, for the ipsilateral monkey (monkey K) that drove the BMI using the cortex ipsilateral to the amputation, the decoder was not based on explicit movement of the intact limb (contralateral limb), but we cannot completely discount the possibility of any imagined or overt movement attempts during BMI learning.

Over the first 8 training sessions, the network of the ipsilateral monkey proceeded to prune its connections and then re-establish a dense network structure. One might speculate that the existing network structure that had controlled the intact limb had to be modified to control the BMI, thus resulting in a transient pruning of connections. However, a curious fact is that the re-establishment of a dense network began at roughly day 30 exactly when the ipsilateral monkey began performing poorly for a short while before returning to her minimum time to success (Fig. 1a). This suggests a novel network structure began to be established on day 30 associated with a new strategy (i.e., different from her original BMI control strategy) for BMI control resulting in a temporary worsening of performance. Although we did not track movements in the intact limb, an intriguing possibility is that the transient pruning and following network expansion may have subtly affected the behavior of the intact limb.

The overall dynamics of network connectivity were quite different between the contralateral and ipsilateral monkeys, but, there were several common features in the nature of their connectivity and its relationship with mean firing rate. First, the excitatory connection density was consistently stronger by about 50–200% than the inhibitory density when considering the entire network. Second, excitatory connection density was generally stronger within the reach and grasp clusters than between the reach and grasp clusters (Fig. 4) indicating that neurons driving a single control dimension were more tightly coupled than neurons across the two control dimensions which could move more independently. In contrast, inhibitory connection density was stronger across the two clusters than within the clusters again suggesting less synchronous movement of the reach and grasp control dimensions.

Monkeys Z and K were two months old when they were amputated, and their corticospinal pathways, especially the cortico-motoneuronal projections, would have needed 6–8 months to fully develop^37, 38^. This provided a unique platform to study network dynamics in an ensemble of neurons that had not completely matured to control the upper limb. This adds to previous BMI work using subjects with pathological conditions. A study using a rat stroke model showed that perilesional cortex (PLC) could be used for BMI control^39^. In human subjects with spinal cord injury (SCI), direct cortical control was also shown to be feasible^40, 41^. With PLCs, the downstream circuits remain intact, and for SCI, depending on the extent of the injury, residual afferent and even efferent pathways may be intact. Our chronic amputee model extends previous studies by showing that neuroprosthetic control and functional plasticity are possible using cortex that had lost both afferent and efferent connections to the missing limb segments years prior to the BMI experiments.

The temporal evolution of mean firing rate led the dynamics of connectivity by at least one training session indicating that the changes in functional connectivity may have been driven by firing rate modulation. If functional connectivity reflects underlying synaptic connectivity, these results suggest that the animal may be voluntarily modulating inputs to motor cortex to affect changes in firing rate which in turn lead to synaptic plasticity among the neurons controlling the BMI. These observations support the argument that network plasticity and reorganization can play an important role in learning to control a neuroprosthetic device.

## Methods

### Neurophysiology and recordings

**Fig. 6:**
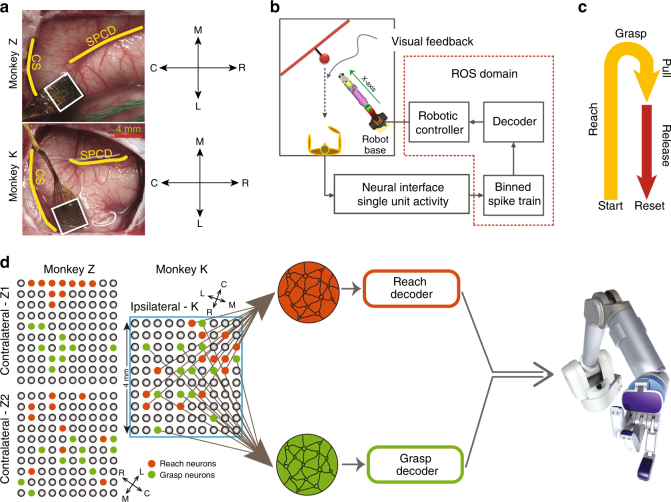
Schematic of the brain–machine interface and the motor task. **a** Cortical landmarks and the location of array implantation (SPCD, superior precentral dimple; CS, central sulcus). **b** Neural spike trains were decoded to generate control signals for the robot. Blocks within the dotted region were implemented in the Robotic Operating System (ROS) platform. **c** The behavioral task comprises reaching toward an object, grasping and pulling it through a partial trajectory back, and release **d** two neural subpopulations, reach (red) and grasp (green) were decoded independently to generate control velocities for the robot. Spatial distribution of the clusters are shown for the contralateral and ipsilateral experiments

Three rhesus (Macaca Mulatta) monkeys (monkeys Z, N and K) with chronic unilateral amputation of the upper limb were used in this experiment. The monkeys had received therapeutic amputations prior to our receiving them for these experiments. The amputations were required to rescue them following injuries to their arm, and were not amputated for the purposes of this experiment. Monkey Z had a transradial amputation and monkeys K and N had elbow disarticulation amputation ~ 10, 9 and 4 years before they arrived to our lab. Monkeys Z and K were 2 months old at the time of amputation, and monkey N was 5 years old. Utah multi-electrode arrays (UEAs) (1.0 mm electrode length and 400 µm pitch from Blackrock Microsystems, Inc., Salt Lake City, UT) were implanted in the upper limb region of primary motor cortex (M1) on the contralateral side to the amputation for monkeys Z and N, and on the ipsilateral side to the amputation for monkey K (see Fig. 6a for monkeys Z and K). Prior to implantation, small electrical currents were applied to the motor cortex using a surface stimulator to evoke movements in the residual limb or intact limb contralateral to the side of the implantation. The surgical and behavioral procedures involved in this study were approved by the University of Chicago Institutional Animal Care and Use Committee and conform to the principles outlined in the Guide for the Care and Use of Laboratory Animals.

Neural data recorded as analog signals were amplified, bandpass filtered between 0.3 Hz and 7.5 kHz, and sampled at 30 kHz before digitization. A fourth order high-pass Butterworth filter with a cut-off frequency of 250 Hz was applied before spike detection. Out of a population of more than 100 neurons recorded from monkeys Z and N, and 200 neurons from monkey K, 20 to 30 of the most stable neurons were chosen, for decoding. Single units were isolated manually using an online hoop-based spike sorting algorithm (Blackrock Microsystems, Inc.) and then assigned to the corresponding clusters for decoding. Each waveform was isolated using 3 or 4 hoops that would define a spike template. These hoop definitions were stored and applied again with minor modifications during the consecutive BMI session. The experimenter manually verified for any missing neurons and ensured that the waveforms remained comparable across days. The mean waveforms of neurons used in two of the experiments are shown in Supplementary Figs. 9–12. In theory, units could have been lost and replaced by other units from one day to the next with the same or similar features on the same channel. So, it is not possible to be absolutely sure that we are recording from the same cell.

### BMI and behavioral task

The top-level diagram of the experimental setup is shown in Fig. 6b. Single neuron activities were binned into 50 ms bins, and the spike trains were decoded to estimate reach and grasp velocities. A Null-space controller^42^ was used to map the decoded output into joint-space velocities of a multiple DOF robot. The animals were operantly conditioned with juice reward to control the robot in order to perform a sequential reach-to-grasp task (Fig. 6c). Each trial in the task began with the robot arm positioned 18 centimeters away from the target object by the robot controller. The animal was then free to volitionally move the robot arm and hand in order to reach forward to the target object, grasp the object, pull it back by 5 cm and then release it. The robot comprised a 7 DOF redundant arm (i.e., the Barrett Whole Arm Manipulator, or WAM) with a 4 DOF hand (i.e., the BarrettHand) (Barrett Technology LLC, Newton, MA). The BarrettHand was modified such that one digit opposed the other two so that it represented the thumb and defined an aperture between it and the other two juxtaposed digits. In this study, the task space was limited to two control dimensions. Each control dimension represented a synergy of joint DOFs which corresponded to either (1) moving the hand along the *x* axis in Cartesian coordinates away from and toward the base of the robot or (2) grasping by opening or closing all three digits of the robotic hand simultaneously (Fig. 6d; Supplementary Fig. 1a). The span of the reach dimension was 18 cm and the maximum grasp aperture was 15 cm. And, the task was self-paced without any time constraints. Two distinct subsets of single units from M1, dubbed as reach and grasp clusters, controlled the reach and grasp dimensions, independently. Subsequently, the decoded outputs were translated into joint-space velocities.

### Decoder initialization

With amputee subjects, neural activity cannot be mathematically mapped to innate motor output due to the lack of an intact limb, and, therefore, we used non-biomimetic algorithms to create the mapping. Two different types of fixed decoders (Decoders A & B) with different neurons for each decoder were used for the contralateral monkey Z comprising two separate experiments (“Contralateral-Z1” and “Contralateral-Z2”) separated by a 40 day interval where the monkey was not exposed to the BMI. Only Decoder B was used with the contralateral monkey N (“Contralateral-N”) and ipsilateral monkey K (“Ipsilateral-K”). Each decoder comprised two one-second long (20 taps of 50 ms each) Wiener filters independently controlling the reach and grasp velocities. The decoders were initialized either, (i) using neural activity related to extrinsic behavior on the ipsilateral side (decoder A) or (ii) using intrinsic resting-state neural activity (decoder B).

*Decoder A*. Neural activity from a population of M1 neurons was mapped to two “desired” velocity profile templates (for reach and grasp dimensions, respectively) inspired by the minimum-jerk velocity profiles that characterize biological reaching^43, 44^. Neural activity was sampled while the monkey performed an ipsilateral arm movement, and this activity was mapped to the velocity template of the reach control dimension. For the grasp dimension, neural activity was sampled as the monkey observed pre-programmed grasp trials performed by the robot, relying on observation-based responses in M1^45, 46^.

And, the system of equations,

1 $${\hat{\mathbf y}} = {\mathbf{X}}\beta ,$$

was solved for *β* using a Ridge regression estimator, given by,

2 $$\beta = ({\mathbf{X}}^{\mathrm{T}}{\mathbf{X}} + \lambda {\mathbf{I}})^{ - 1}{\mathbf{X}}^{\mathrm{T}}{\mathbf{y}}{\mathrm{,}}$$

where, ${\hat{\mathbf y}}$ is the decoded velocity for a given neural activity matrix **X** (composed of activity from *n* neurons ×20 bins of 50 ms history each). The ridge parameter $\lambda$ ensures that the inverted matrix has a condition number no larger than 10^3^. Estimators based on ridge regression are known to reduce the variance of the estimate but with a certain bias. This variance-bias trade-off is useful to avoid overfitting of the data, and handles unseen data presented to the estimator.

*Decoder B*. While Decoder A is more suitable for unilateral amputees capable of moving the ipsilateral arm, an unsupervised approach^47^ based on resting-state neural activity was tested that completely relaxes any requirement of motor behavior. This decoder uses spontaneous neural activity characteristics to estimate the decoder coefficients. Tap weights were sampled from a multivariate Gaussian generative model space denoted by,

3 $$\beta \sim N(0,{\mathbf{S}}),$$

where, ${\mathbf{S}}$ represents the sample covariance matrix derived from spontaneous neural activity as,

4 $${\mathbf{S}} = \frac{1}{M}{\hat{\mathbf X}}^{\mathrm{T}}{\hat{\mathbf X}},$$

where, ${\hat{\mathbf X}}$ is the mean-centered neural firing rate matrix **X**, and ***M*** is the number of filter taps. The eigenvectors of **S** span the entire covariance space. They were determined by decomposing the matrix,

5 $${\mathbf{Y}} = \frac{1}{{\sqrt M }}{\hat{\mathbf X}} = {\mathbf{U}}{\mathrm{\Sigma }}{\mathbf{V}}^{\mathrm{T}},$$

The decoder coefficients were estimated using a subset of the eigenvectors ${\mathbf{U}}_{\mathrm{s}} \subset {\mathbf{U}}$, denoted as,

6 $${\mathbf{w}} = {\mathbf{U}}_{\mathrm{s}}\phi ,$$

where, $\phi$ is the vector of spanning coefficients that minimizes the cost function,

7 $$J = \sqrt {{\mathbf{w}}^{\mathrm{T}}{\mathbf{S}}^{ - 1}{\mathbf{w}}} + \left| {\bar z - 1} \right| + c_r\left( {\mathop {\sum}\limits_g {r_g(\mu _g,\sigma _g^2)} + \mathop {\sum}\limits_g {c_s.r_s(\gamma _g)} } \right) + \left| {1 - f} \right|,$$

where, $\sqrt {{\mathbf{w}}^{\mathrm{T}}{\mathbf{S}}^{ - 1}{\mathbf{w}}}$ is the Mahalanobis distance to the distribution assumed in Equation-3, $\left| {\bar z - 1} \right|$ is the term used to reduce output fluctuations by decreasing the number of zero-crossings of the zero-mean decoded output, $r_g(\mu _g,\sigma _g^2)$ (Rao score) and $r_s(\gamma _g)$ ensure Gaussian properties in the decoded output. The final term, $\left| {1 - f} \right|$ ensures a more equitable distribution of labor across all the neurons in use, where *f* denotes the fraction of neurons contributing to 90% of the decoded output.

Each monkey was assigned two clusters ({reach, grasp}) to control the robot. The decoders used {15, 15}, {9, 9} and {19, 10} pairs of neurons for the reach and grasp clusters from Monkeys K, N and Z, respectively. After n-hours of training, Monkey Z was reassigned with new clusters with {10, 10} neurons and trained on the same behavioral task. Monkey K used the ipsilateral side of the amputation to control the robot, while Monkeys N and Z used the contralateral implantations.

### Neural clusters

Before running the BMI experiments, we assessed the stability of neurons by tracking them over 7 sessions. Based on prior published work from our lab^48^, we have found that neurons that are stable over 7 sessions are highly likely to remain stable over the next session. Therefore, we chose a subset of neurons for BMI control that had remained stable for 7 sessions prior to the BMI experiment. These stable units were clustered into distinct groups based on strong correlated firing in the “resting” state, while the animal was sitting idly in its chair^49^. Units’ identities were tracked using their waveform profiles^50^ prior to assessing their stability. Instead of using all available neurons for each control dimension, we chose to assign a particular cluster of highly correlated neurons to control a single control dimension. This is due to the incremental approach we used for BMI exposure such that the monkey was first exposed to one control dimension (i.e., reach) and then given the grasp dimension to control together with the previous dimension. Thus, the monkey could, in principle, learn to modulate one cluster to control the reach and then learn to modulate an independent cluster of neurons to control the grasp instead of relearning to modulate the entire neural population for reach and grasp. Two of the clusters were arbitrarily chosen to control the reach and grasp control dimensions. The analyses presented here are from when the monkeys were exposed to using both clusters. Monkey Z used 29 neurons {19 reach, 10 grasp} for Decoder A, and 20 neurons {10 reach, 10 grasp} for Decoder B. Likewise, 18 neurons {9 reach, 9 grasp} were used for Decoder B with monkey N. Finally, for monkey K, 30 neurons {15 reach, 15 grasp} were used for decoding. Decoder B used clusters with balanced number of neurons in each cluster (see Table 2 for the balancing algorithm). We also reserved other recorded neurons that were stable and had resting-state characteristics that were similar to neurons in the clusters, to be used as replacement if neurons were lost. However, in the experiments reported here, we did not lose any neurons.

**Table 2:** Algorithm for balancing units per cluster

**Require**: A set of clusters of stable units, C, and a minimum number of units per cluster, *M*
**Ensure**: At least *M* stable units are assigned per cluster
N ← 0
^ **for all***c* ∈ C **do**^
^ *N ← N* + (*M* − sizeof(*c*))^
**end for**
**while** *N* > 0 **do**
Identify all available donorClusters with size of (*c*)>M
**for all** *c* ∈ donorClusters **do**
Find the electrode *e*_c_ that has the most weakly connected stable units
**end for**
Pick a *donorCluster* ← c such that c ← arg min_*i∈*donorClusters_ funcon (*i*, *e*_*i*_)
Assign all units on *e*_*c*_ to a *r* *eceivingCluster* ← υ such that υ ← arg max_*i*∈*C*_ funcon (*i*, *e*_*i*_)
N ← 0
**for all** *c* ∈ C **do**
*N* ← *N* + (*M*−size of (*c*))
**end for**
**end while**

### Directed effective connectivity

We used a Granger-type causality estimation technique generalized for point-processes to infer directed effective connectivity and its polarity (i.e., excitatory or inhibitory) among simultaneously recorded neurons^51, 52^ Neural activity epochs during specific periods of the decoded velocity were used to infer causality. The magnitude of decoded velocities were normalized to range between 0 and 1, and periods were chosen when the velocities were ≤0.05 for 500 ms followed by a transition to ≥0.5 within 50 ms and then maintained ≥0.5 for another 500 ms. The instantaneous spiking of a candidate neuron was characterized as its conditional intensity function (CIF), λ(t|H(t)), where *H(t)* denotes the spiking history of all neurons in the ensemble up to time t. The log CIF of each neuron was modeled as a linear combination of its covariates, i.e., *H(t)*, with a model order (*M*) of 60 ms and a bin size (*W*) of 3 ms (i.e. 20 spike history terms per source neuron). The model order, i.e., the history for each neuron, was optimized using AIC (Akaike’s information criterion). Then, to determine the influence of a source neuron on the candidate neuron, the log-likelihood ratio between models (i) including and (ii) excluding the history of the source neuron was computed. If the model performance decreased significantly (*p* < 0.05, *χ* ^2^-test, False Detection Rate corrected for multiple comparison) when the source neuron was excluded from the model, we inferred that the source neuron was effectively connected to the candidate neuron. Finally, the sign of the sum of corresponding spike history coefficients was used to determine whether the directed connection was excitatory or inhibitory. We assessed the consistency of polarity of the history terms by computing the ratio between the number of history terms with dominant polarity and the number of terms with non-dominant polarity for each neuron within a session. Supplementary Fig. 13 shows the distribution of this ratio for each individual session. The modes of these distributions are approximately at a ratio of 2 indicating that a large portion of connections emanating from a neuron have twice as many history terms of one particular sign. The implementation steps for causality estimation are provided in Table 1.

### Data availability

The data that support the findings of this study are available from the corresponding authors upon reasonable request.

## Electronic supplementary material

Supplementary Information

Description of Additional Supplementary Information

Supplementary Movie 1

Supplementary Movie 2
