# Supplementary material for: Changes in cortical network connectivity with long-term brain-machine interface exposure after chronic amputation
Source: Nat Commun. 2017 Nov 27;8:1796. doi: 10.1038/s41467-017-01909-2 (PMC5703974; doi:10.1038/s41467-017-01909-2)
Supplement: Supplementary file 1 — Supplementary Information [file 41467_2017_1909_MOESM1_ESM.pdf]

Supplementary Figures

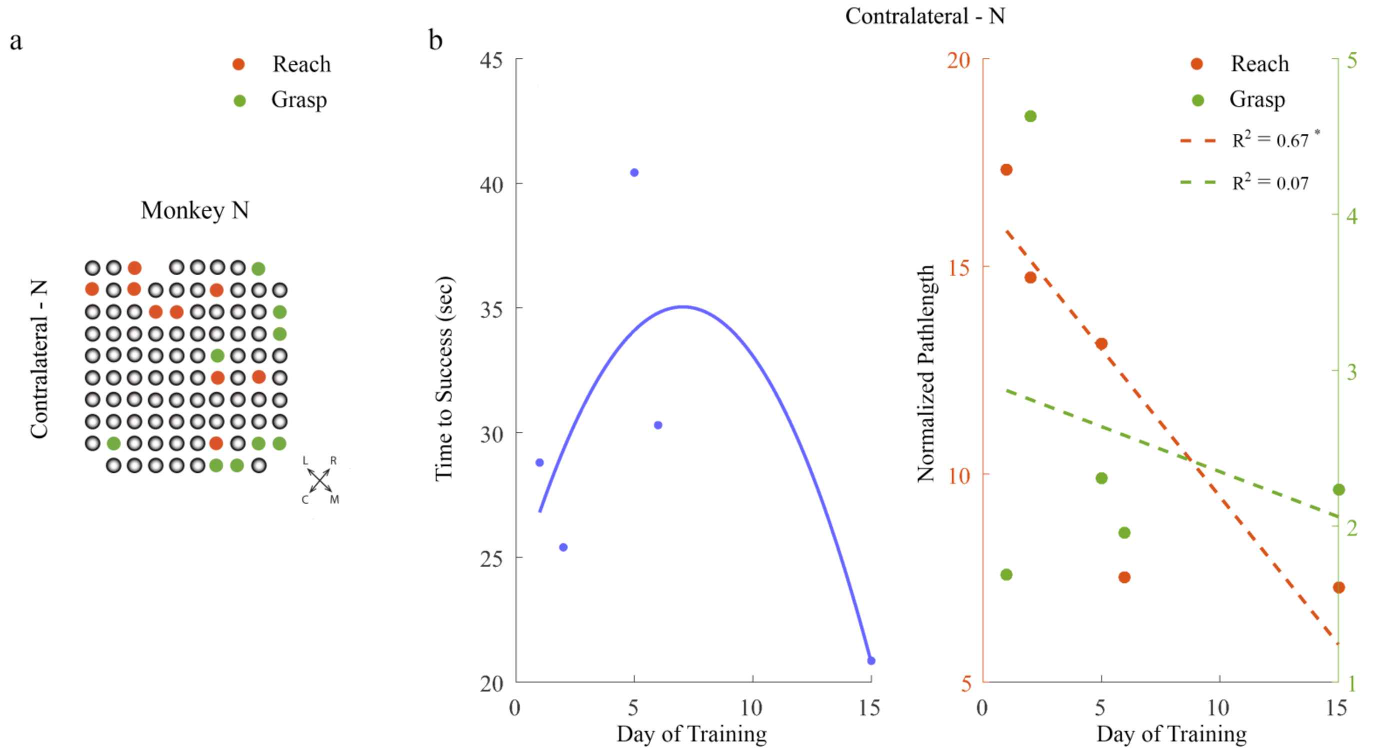

Supplementary Fig. 1: Neural clusters and behavioral performance of monkey N (Contralateral-N) (a) shows the reach and grasp clusters used for decoding in the Contralateral-N experiments. (b) shows the behavioral performance.

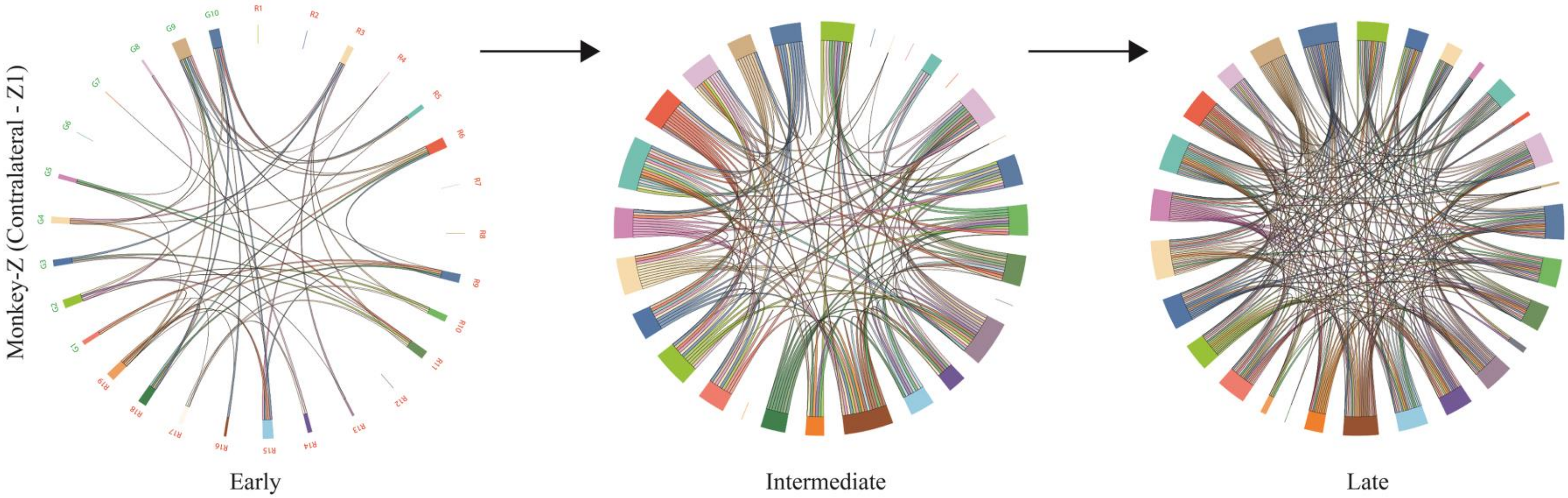

Supplementary Fig. 2: Network density in the neuronal populations. Three instances along the days of training showing monotonic increase in network connectivity in the Contralateral-Z1 experiment. A total of 29 neurons used in the decoding is shown here and the trend was similar to the Contralateral-Z2 experiment.

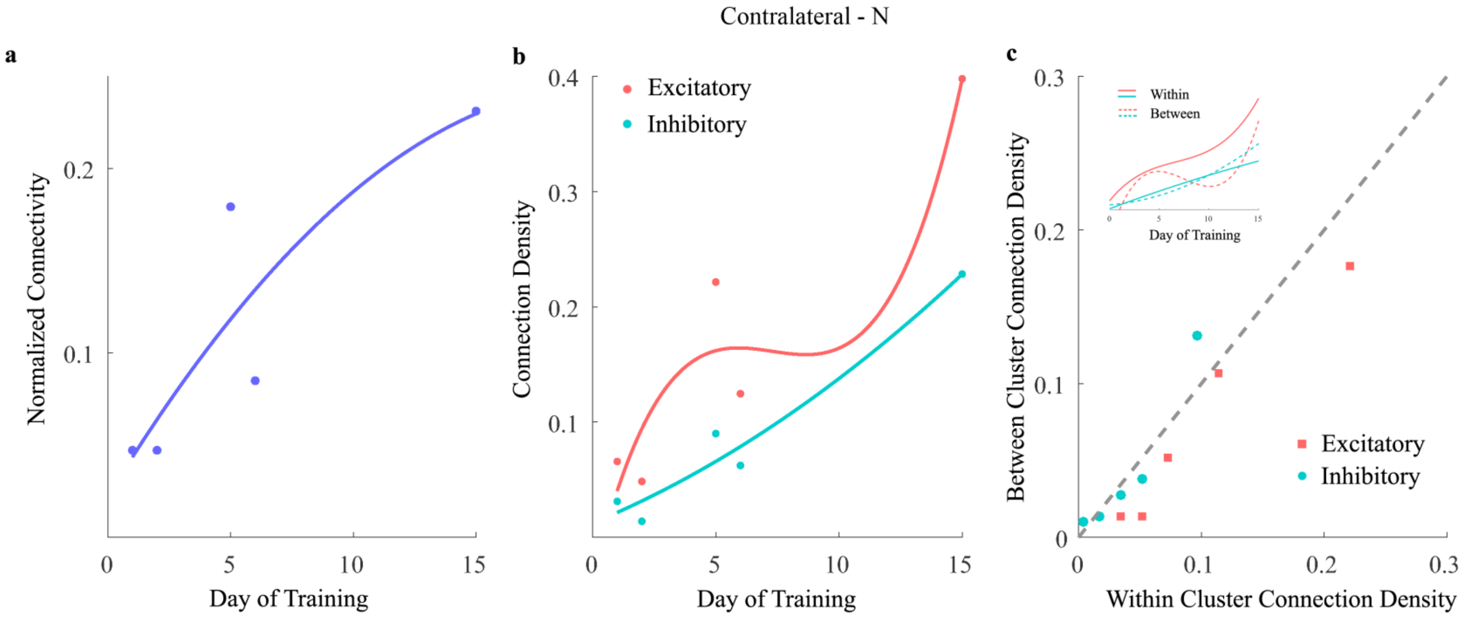

Supplementary Fig. 3: Network connection density in the ensemble from the Contralateral-N monkey. (a) shows the change in overall network density over the course of training, (b) shows the network resolved into excitatory and inhibitory connections, (c) compares the density within and across the clusters.

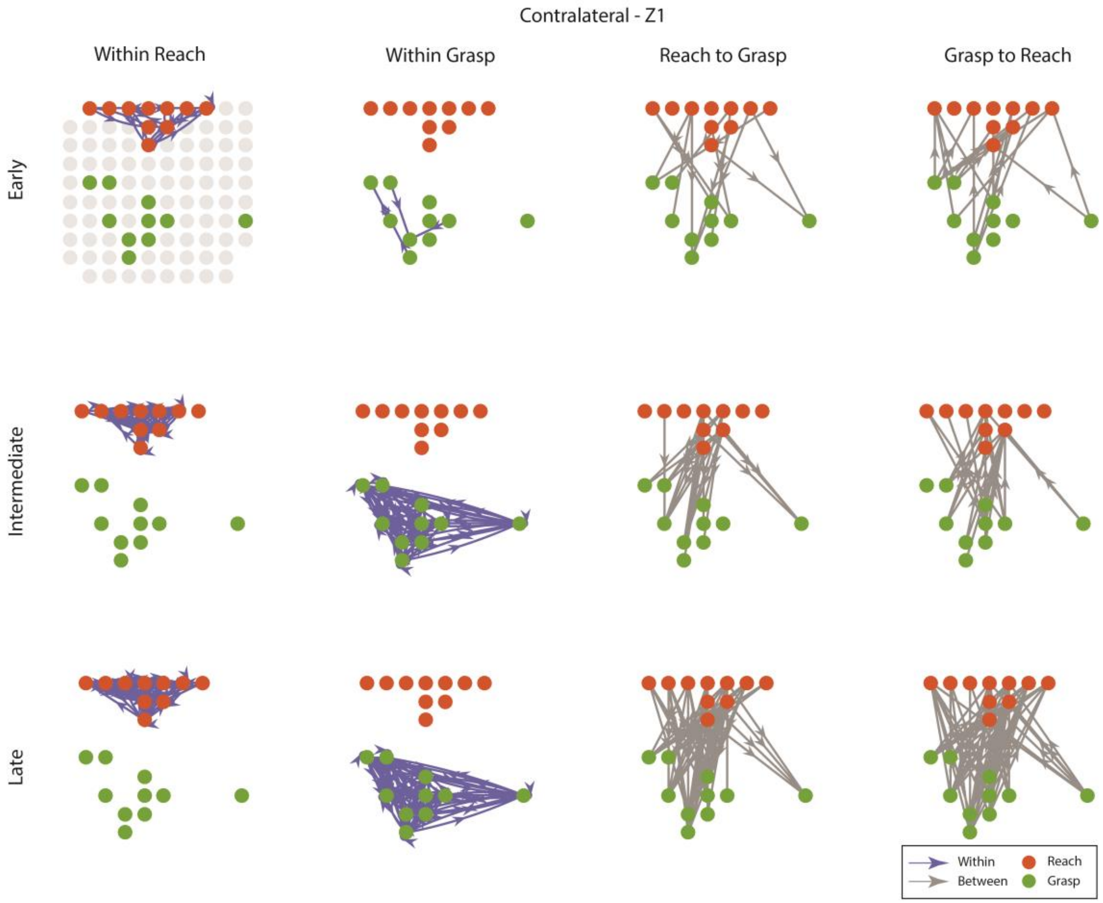

Supplementary Fig. 4: Directionality of the network in the reach and grasp clusters. The connectivity resolved as within the reach and grasp clusters, projections from grasp to the reach neurons and vice versa is shown for the contralateral Z1 experiments.

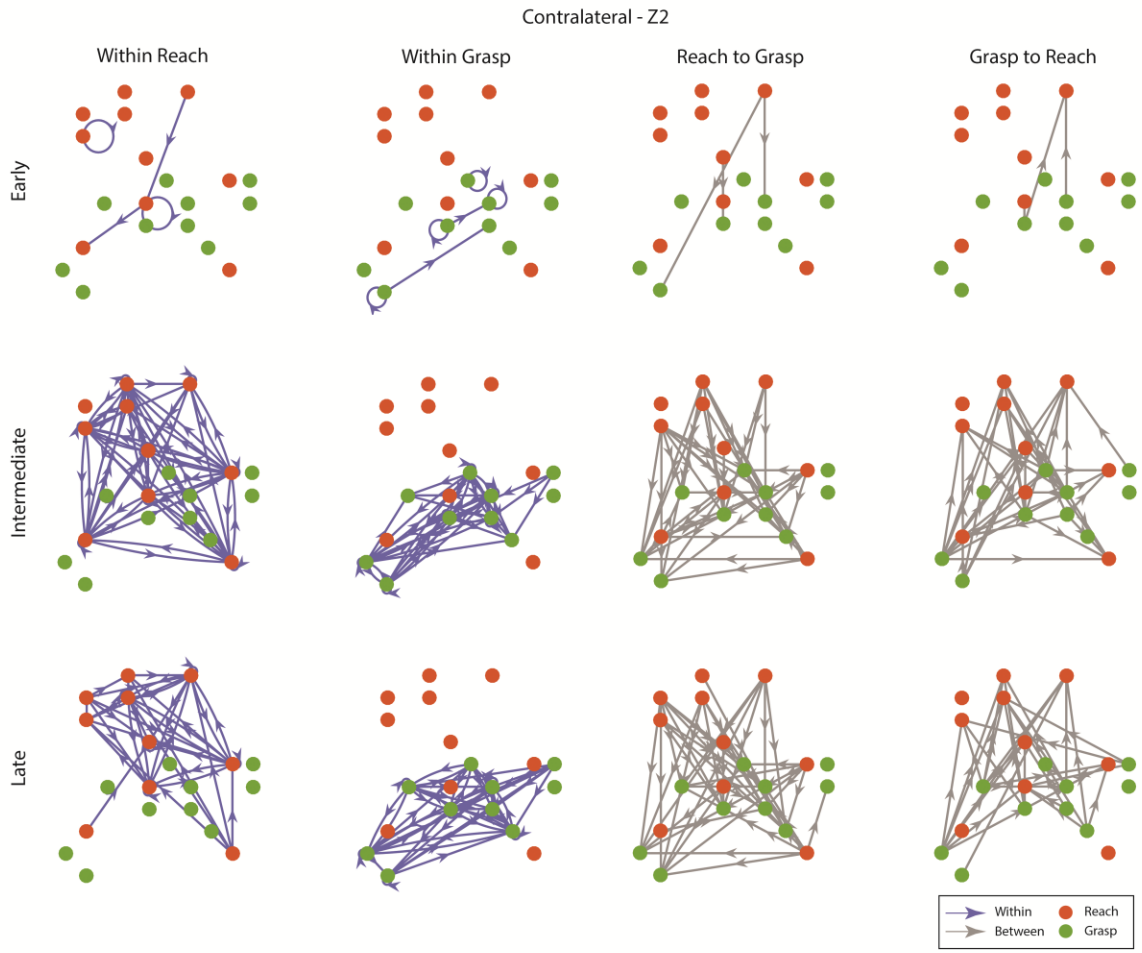

Supplementary Fig. 5: Directionality of the network in the reach and grasp clusters. The connectivity resolved as within the reach and grasp clusters, projections from grasp to the reach neurons and vice versa is shown for the contralateral Z2 experiments.

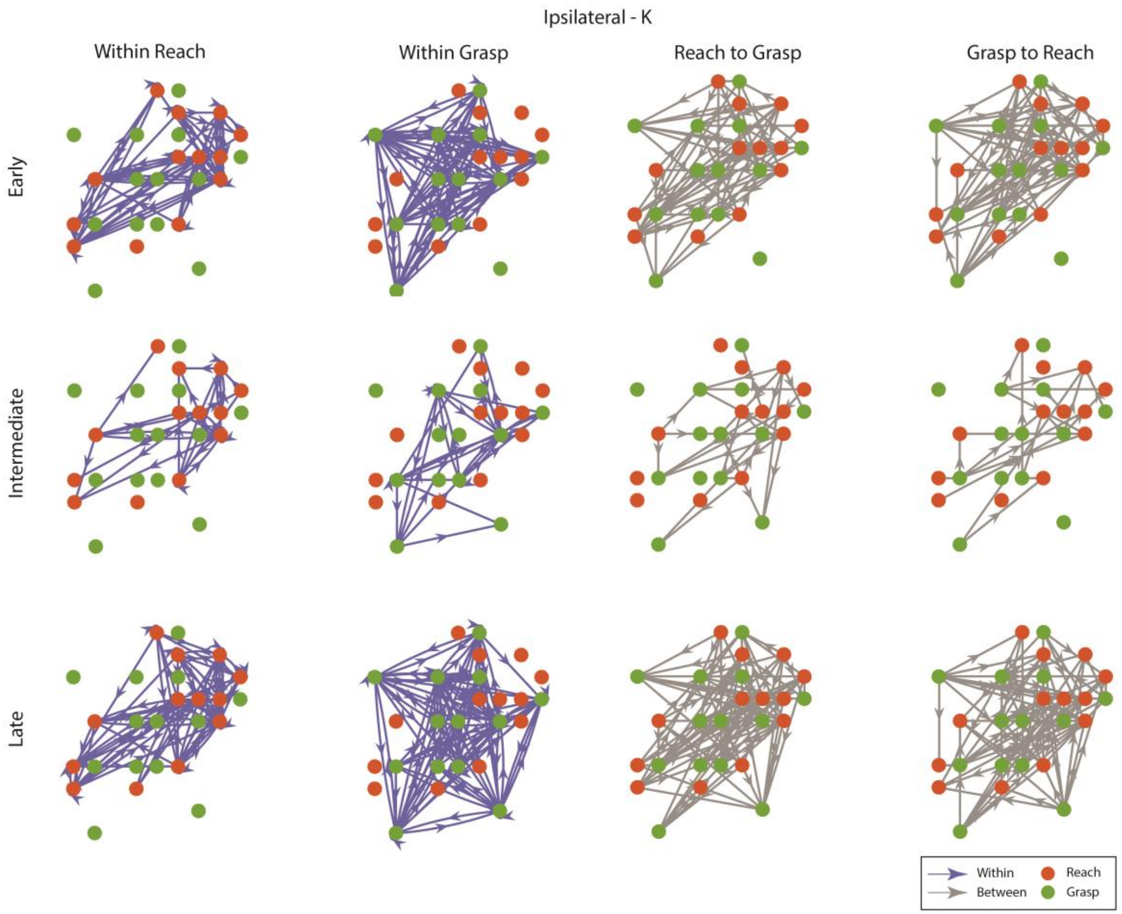

Supplementary Fig. 6: Directionality of the network in the reach and grasp clusters. The connectivity resolved as within the reach and grasp clusters, projections from grasp to the reach neurons and vice versa is shown for the ipsilateral K experiments.

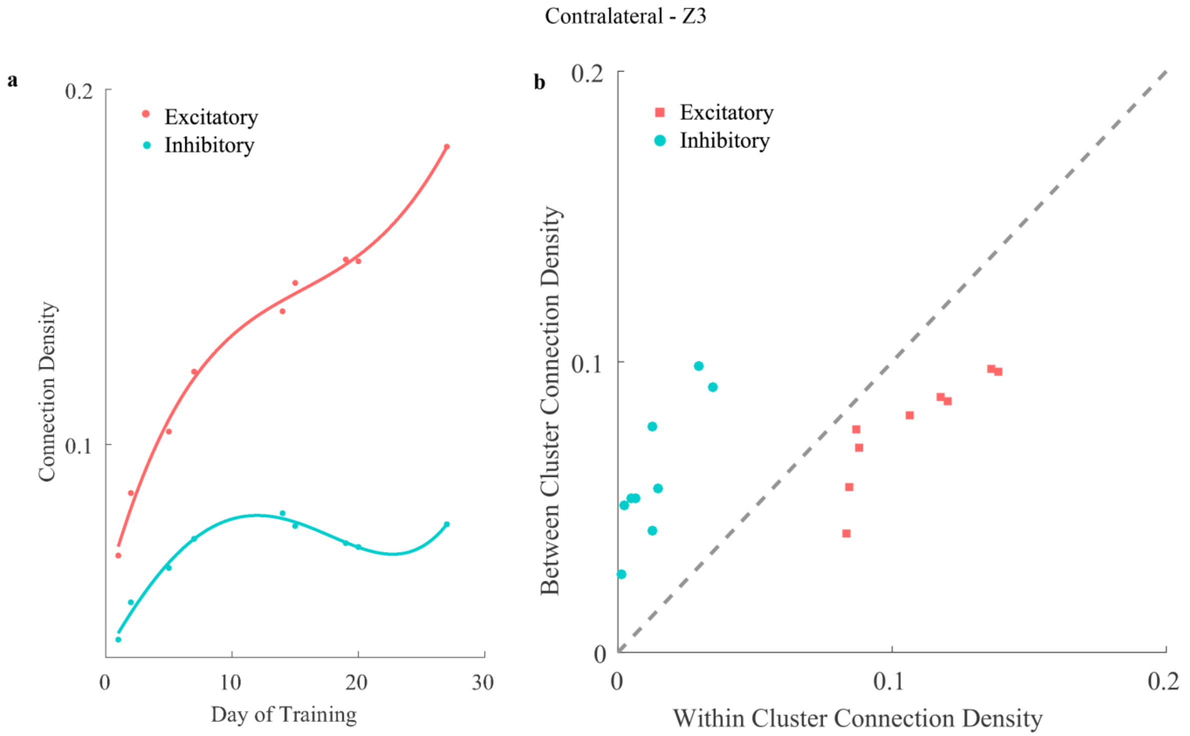

Supplementary Fig. 7: Network dynamics in the ensemble from Contralateral monkey Z using a position controller (Contralateral-Z3). The animal used the same velocity decoder (Decoder-A) that was used in Contralateral-Z1 experiments, but here the decoded velocities were integrated to generate position commands for the robot. The two sets of experiments (Contralateral Z1 and Z3) were separated by a period of ~50 days. (a) shows the change in excitatory and inhibitory connections over the course of training, (b) compares the density within and across the clusters.

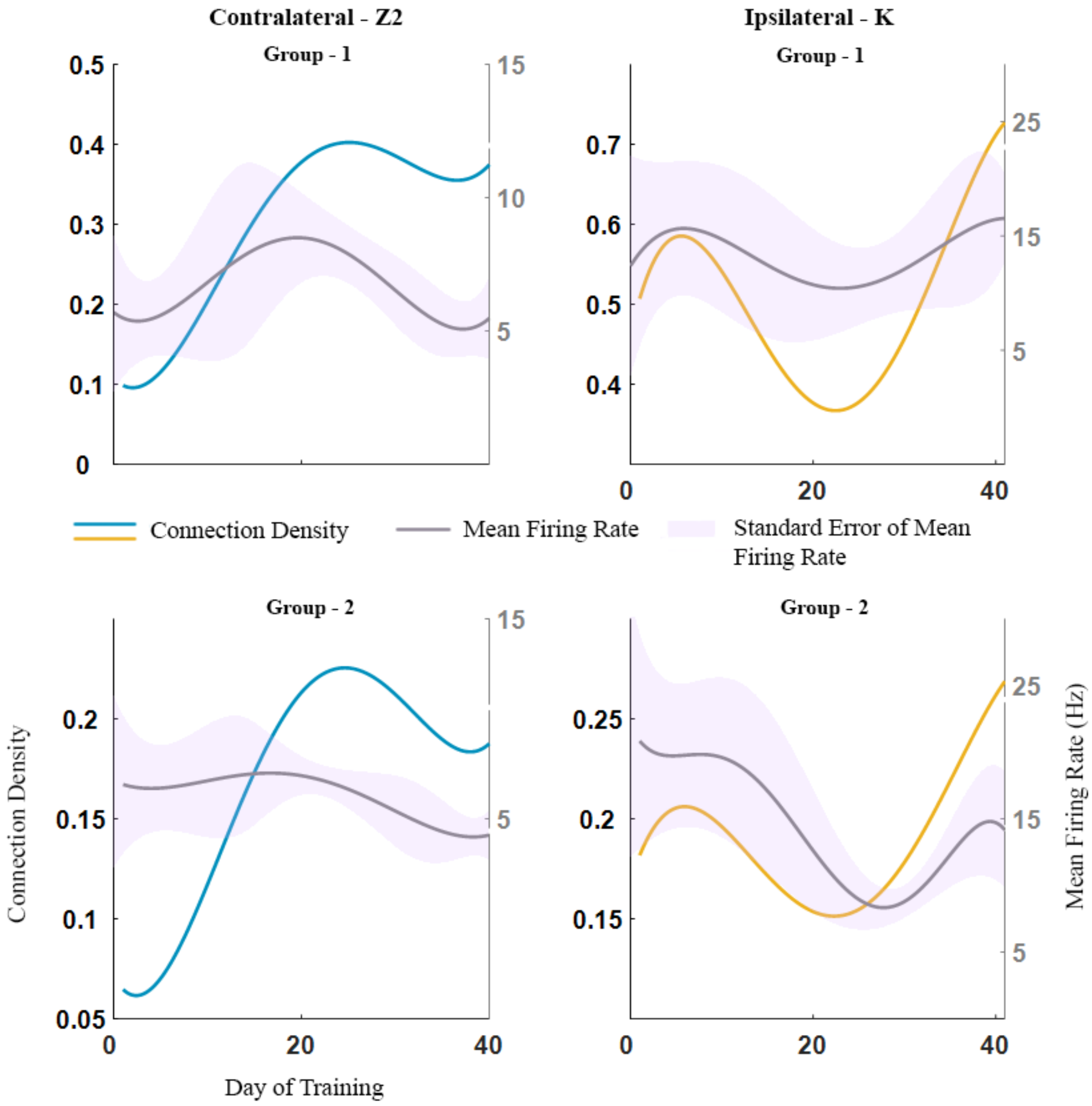

Supplementary Fig. 8: Analysis of effect of firing rate on network connectivity in a subset of neurons. Neurons within the ensemble were classified into two groups based on the presence or absence of significant correlation between their firing rates and the network density. The panels on the left show the network dynamics of these groups in the contralateral-Z2 monkey and the panels on the right corresponds to the ipsilateral-K monkey. Panels on the top row correspond to neurons that showed significant correlation with firing rate and the panels in the bottom row show the connection density in neurons that showed non-significant correlation with the firing rate.

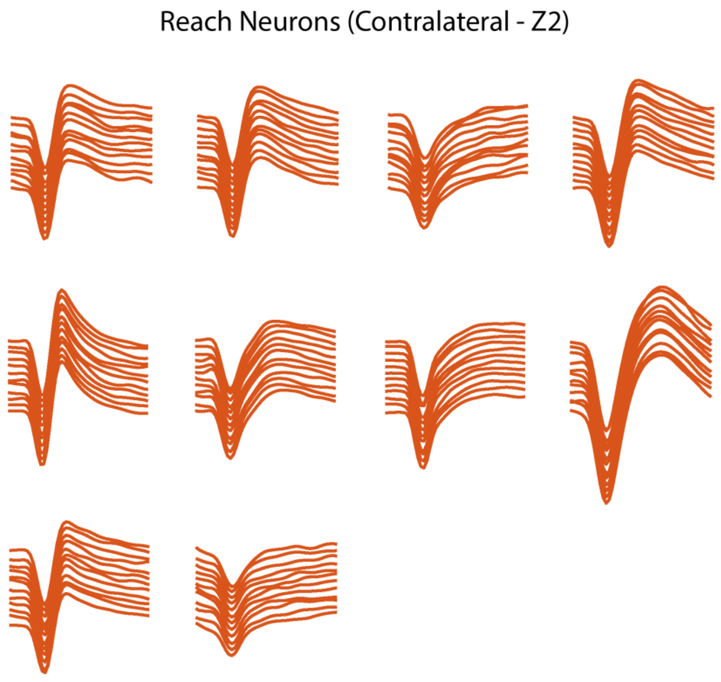

Supplementary Fig. 9: Mean waveform of neurons in the reach cluster from contralateral monkey Z. The neurons shown here were assigned to the reach cluster in the Contralateral-Z2 experiments. The traces are from all of the 12 BMI sessions corresponding to Contralateral-Z2 experiments. Each trace represents the mean waveform from a single recording session.

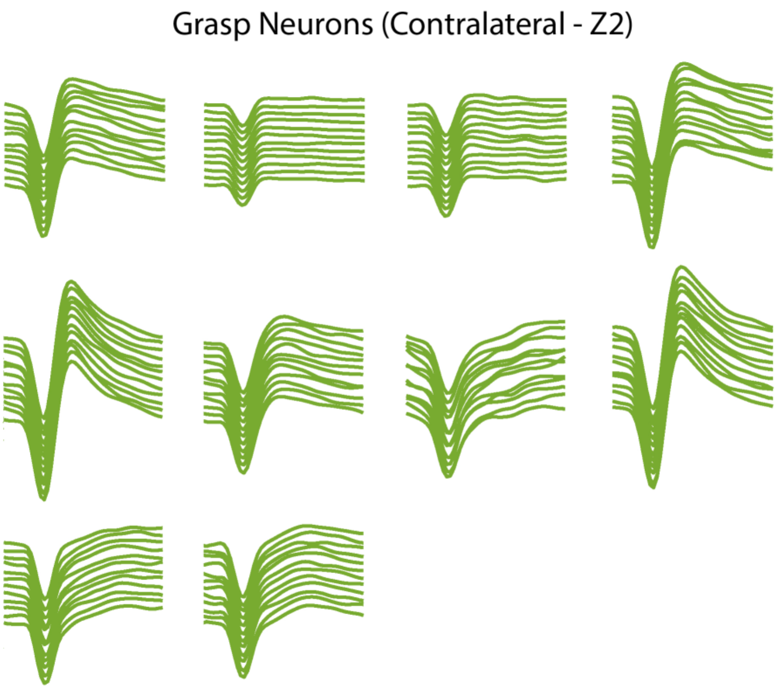

Supplementary Fig. 10: Mean waveform of neurons in the grasp cluster from contralateral monkey Z. The neurons shown here were assigned to the grasp cluster in the Contralateral-Z2 experiments. The traces are from all of the 12 BMI sessions corresponding to Contralateral-Z2 experiments. Each trace represents the mean waveform from a single recording session.

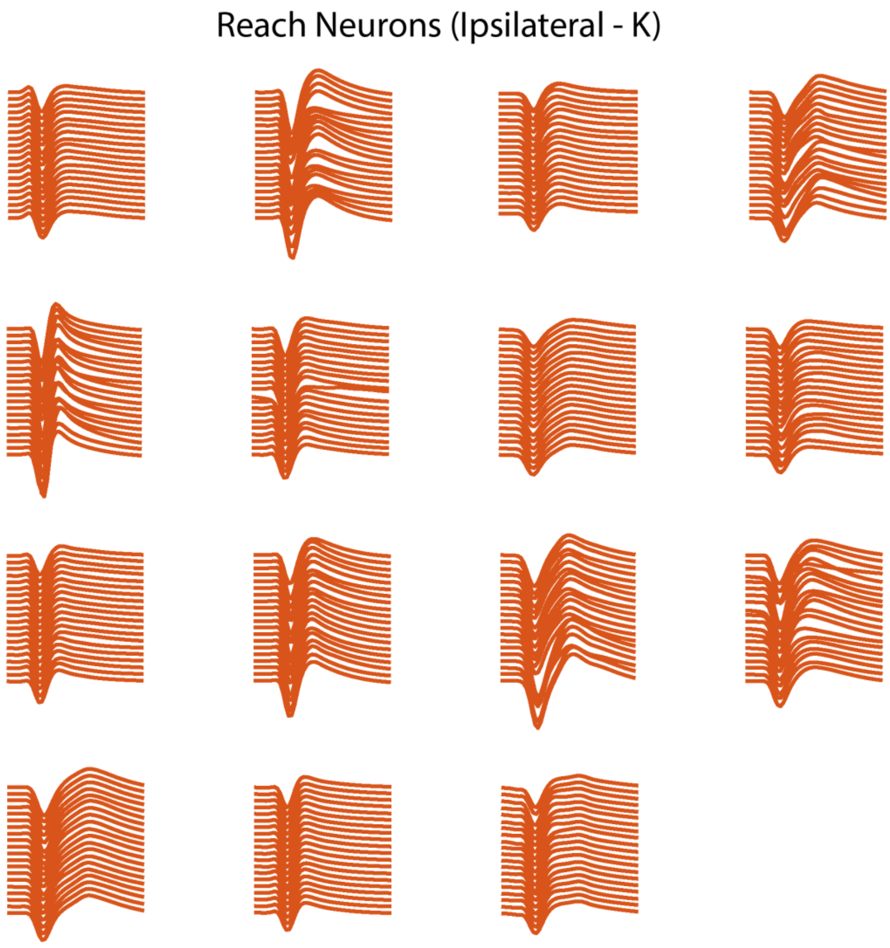

Supplementary Fig. 11: Mean waveform of neurons in the reach cluster from ipsilateral monkey K. The neurons shown here were assigned to the grasp cluster in the Ipsilateral-K experiments. The traces are from all of the 20 BMI sessions corresponding to Ipsilateral-K experiments. Each trace represents the mean waveform from a single recording session.

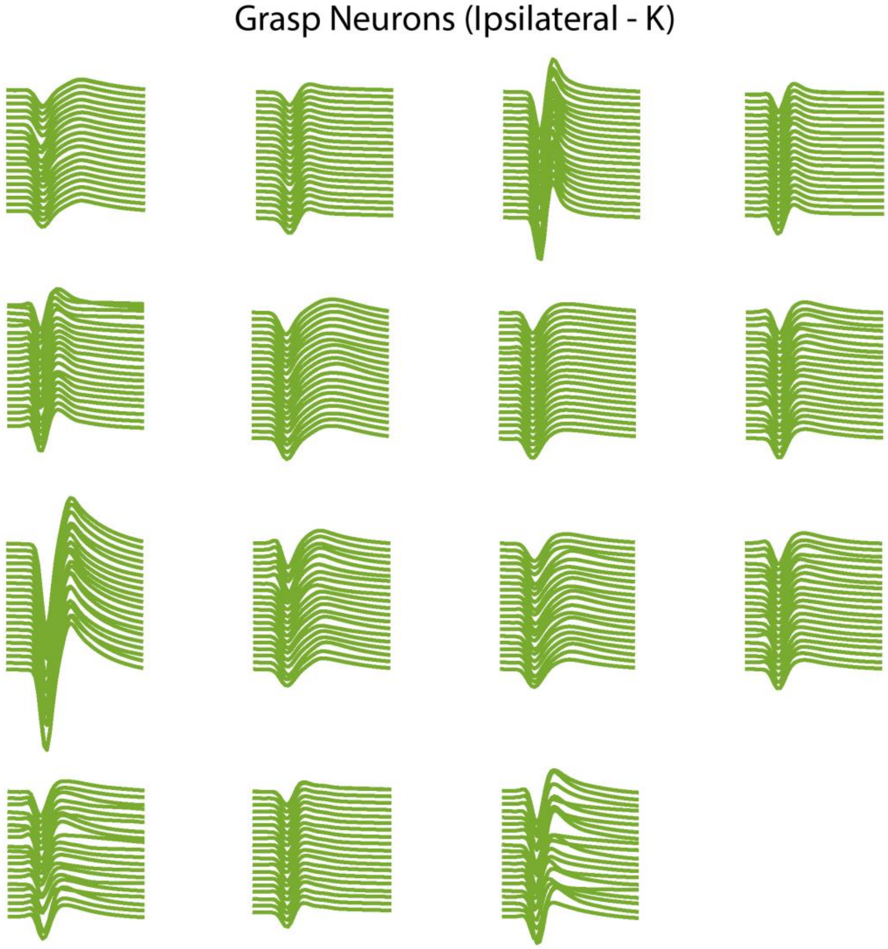

Supplementary Fig. 12 Mean waveform of neurons in the grasp cluster from ipsilateral monkey K. The neurons shown here were assigned to the grasp cluster in the Ipsilateral-K experiments. The traces are from all of the 20 BMI sessions corresponding to Ipsilateral-K experiments. Each trace represents the mean waveform from a single recording session.

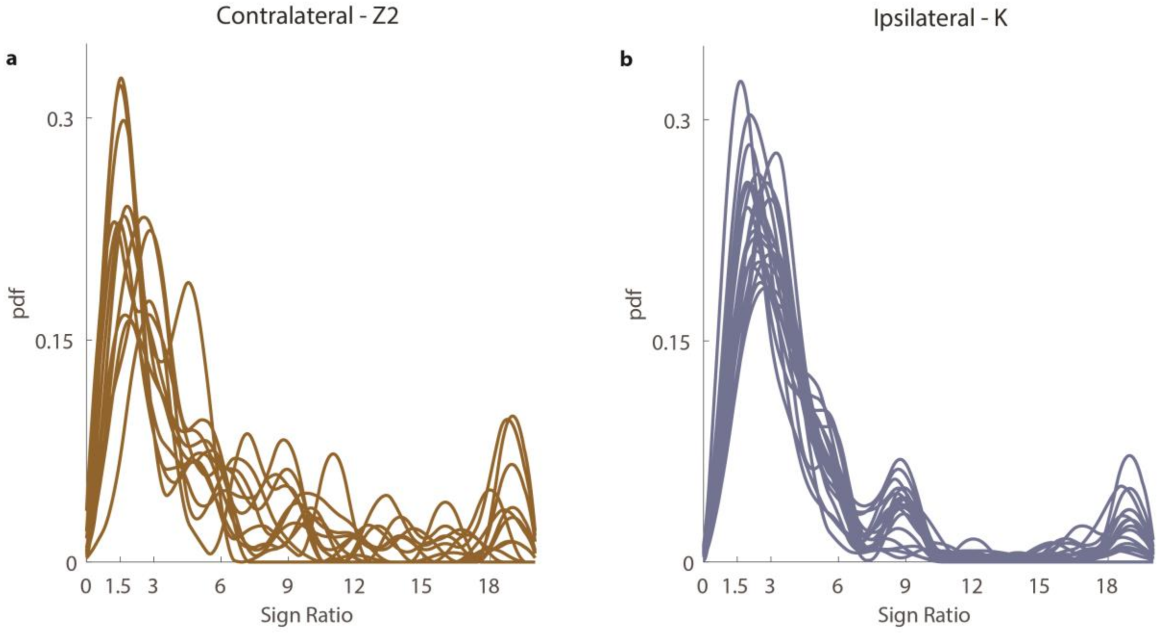

Supplementary Fig. 13: Consistency of polarity in the network model. Sign ratio is defined as the ratio between the number of history terms with dominant polarity and the number of terms with non-dominant polarity. (a) shows the distribution of sign ratios of the contralateral-Z2 model and (b) shows the distribution of sign ratio traces for the ipsilateral K model. Each trace corresponds to one experimental session. A large portion of the coefficients have at least 2/3rd majority of one particular sign through the history of bins.
