# Supplementary material for: Changes in cortical network connectivity with long-term brain-machine interface exposure after chronic amputation
Source: Nat Commun. 2017 Nov 27;8:1796. doi: 10.1038/s41467-017-01909-2 (PMC5703974; doi:10.1038/s41467-017-01909-2)
Supplement: Supplementary file 2 — Description of Additional Supplementary Information [file 41467_2017_1909_MOESM2_ESM.pdf]

Description of Additional Supplementary Files

File Name: Supplementary Movie 1
Description: Qualitative demonstration of Monkey Z (contralateral) controlling a multi-degree-of-freedom robot through a BMI

File Name: Supplementary Movie 2
Description: Qualitative performance comparing early and late BMI exposures in Monkey Z (contralateral)
